# Pulmonary plasma cell disorders: histopathology, diagnosis, and clinical perspectives

**DOI:** 10.1007/s00428-025-04213-3

**Published:** 2025-08-23

**Authors:** Federica Pezzuto, Giordano Fiorentù, Gioele Castelli, Simone Zanella, Chiara Giraudo, Marianerica Tinè, Fiorella Calabrese

**Affiliations:** Department of cardiac, thoracic, vascular sciences and public health, University of Padova, Padua, 35121 Italy

**Keywords:** Plasma cell disorders, IgG4-related disease, Multiple myeloma, Plasmacytoma, Light chain deposition disease, Lung diseases

## Abstract

Plasma cell disorders in the lungs include a broad range of conditions from benign reactive processes to malignant neoplasms. This article provides a comprehensive analysis of these diseases, focusing on their histopathological, immunohistochemical, and diagnostic characteristics. Four case studies illustrate the diagnostic challenges in distinguishing between reactive plasma cell infiltrates and neoplastic conditions. Key diagnostic tools, such as histological analysis and molecular techniques, are discussed to guide clinical evaluation. A systematic approach to diagnosis and management is mandatory for treatment decisions. Understanding the full spectrum of plasma cell abnormalities in the lung enables clinicians and pathologists to achieve accurate diagnoses and improve patient outcomes.

## Introduction

Plasma cell-associated lung disorders represent a diagnostically challenging spectrum of conditions, ranging from benign reactive infiltrates to malignant neoplasms [1]. This article provides an in-depth analysis of plasma cell-related lung disorders, emphasizing the clinical, radiological, and histopathological features that are crucial for a precise diagnosis. By integrating real-world case studies with advanced diagnostic approaches, this review offers a comprehensive guide to enhancing diagnostic accuracy and improving patient care. The aim is to bridge the gap between traditional diagnostic techniques and clinically applicable ancillary tools, including immunohistochemistry (IHC), flow cytometry, light chain studies, and molecular assays such as mass spectrometry and immunoglobulin heavy chain (IGH) rearrangement, offering novel insights into the management of complex plasma cell conditions in the lungs.

## Case description

### Case 1

**Fig. 1:**
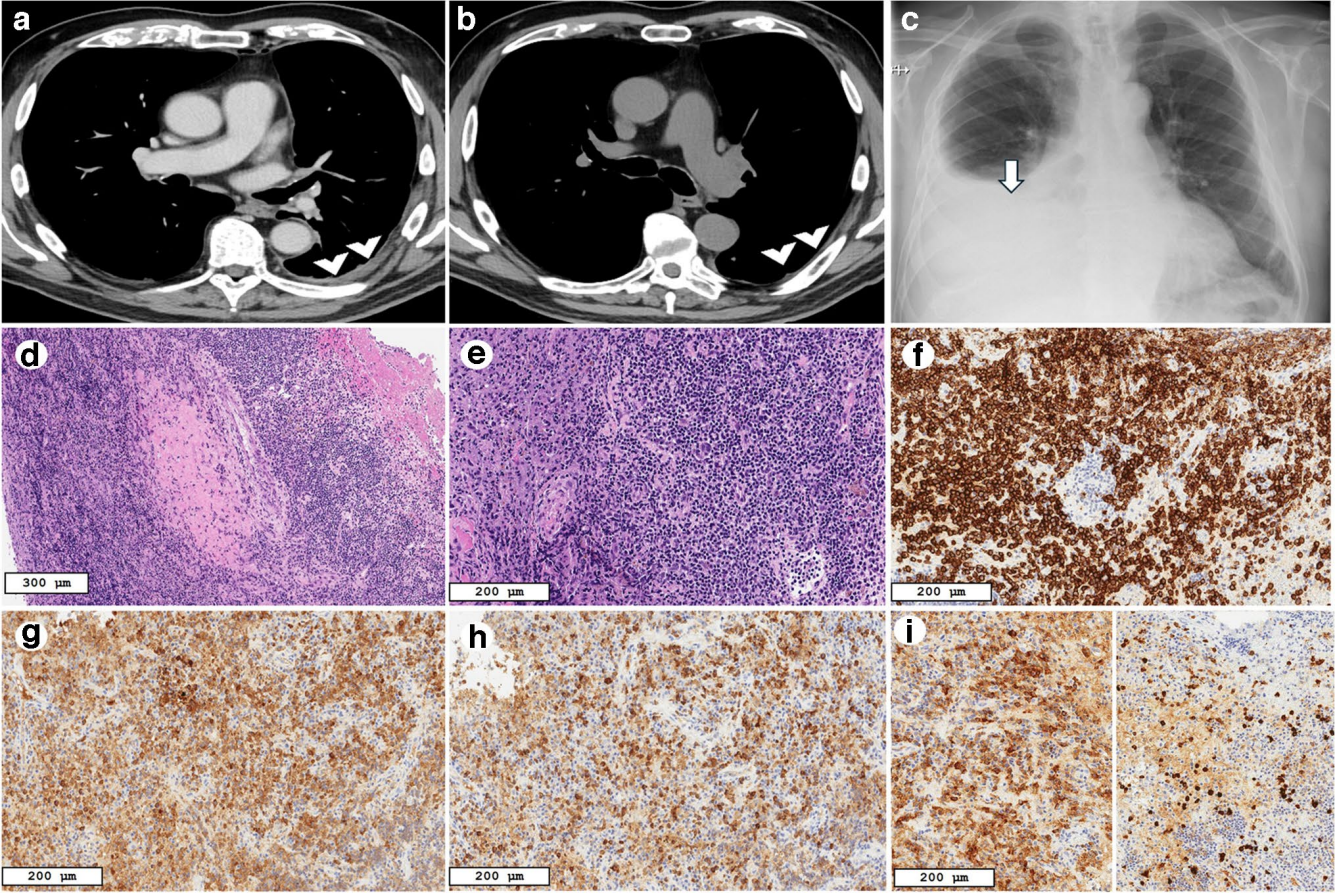
Case 1: Radiological and histopathological features of IgG4-related pleural disease. The chest CT scan with contrast medium injection performed after drainage of the pleural effusion showed pleural thickening in the lower lobe (white arrowheads in **a**). The CT scan performed after 6 months demonstrated a full recovery (white arrowheads in **b**). One year later, the chest radiograph showed a right pleural effusion (white arrow in **c**). Hematoxylin–eosin staining highlighted the overall inflammatory pattern, with storiform fibrosis (**d**, **e**, scale bar: 300 µm). Lymphoid markers showed a prominent plasma cell component (CD138, **f**, scale bar: 200 µm). Light chain restriction analysis demonstrated polyclonality with both kappa (**g**, scale bar: 200 µm) and lambda (**h**, scale bar: 200 µm) expression. IgG4 immunostaining revealed an increased number of IgG4-positive plasma cells (85 per HPF, IgG/IgG4 ratio: 60%) (**i**, scale bar: 200 µm, left side: IgG immunostaining, right side IgG4 immunostaining)

A 79-year-old non-smoker with a history of hypertensive heart disease, hyperuricemia, and type II diabetes was referred to our pulmonology unit after experiencing dyspnea and cough following the second dose of ChAdOx1 nCoV-19 SARS-CoV-2 vaccine. Laboratory tests revealed mild eosinophilia and lymphopenia, elevated lactate dehydrogenase, and normal C-reactive protein levels. He underwent a chest X-ray that demonstrated extensive pleural effusion and atelectasis on the left side. After pleural drainage, a computed tomography (CT) scan was performed which showed pleural thickening (Fig. 1a) and a few small reactive mediastinal lymph nodes. The pleural effusion had exudative features. A medical thoracoscopy was performed. The pleural biopsies were characterized by the presence of lymphoid follicles with germinal centers and lymphoplasmacytic infiltration, with no immunophenotypic aberration. Chronic lymphocytic follicular pleuritis was diagnosed. Autoimmune markers, except for rheumatoid factors, were negative. After closely monitoring the patient, a follow-up CT scan at 6 months showed full recovery (Fig. 1b). One year later, the patient was hospitalized with a right pleural effusion (Fig. 1c), elevated C-reactive protein, persistent lymphopenia, and positive rheumatoid factor. Autoimmune screening detected increased anti Rib-P40 and U1RNP levels. A second medical thoracoscopy was performed. The pleura appeared macroscopically inflamed with regions of whitish parietal pleura alternating with others showing abnormal vascularization. The histopathological analysis revealed massive pleural inflammation with increased lymphocytic and plasma cell components, with scattered eosinophils and small foci of necrosis and stromal fibrosclerosis (Fig. 1d, e). The number of IgG4-positive plasma cells and the IgG/IgG4 ratio were increased, highly suggestive of an IgG4-related disease (RD) (Fig. 1f–i). The serum IgG4 level was elevated at 185 mg/Dl, while no other organ involvement was identified clinically. After the rheumatologist consultation, the final diagnosis of IgG4-RD was achieved, in line with the international criteria [2]. Treatment with oral steroids started, with progressive clinical improvement and no evidence of recurrence at thoracic ultrasounds.

### Case 2

**Fig. 2:**
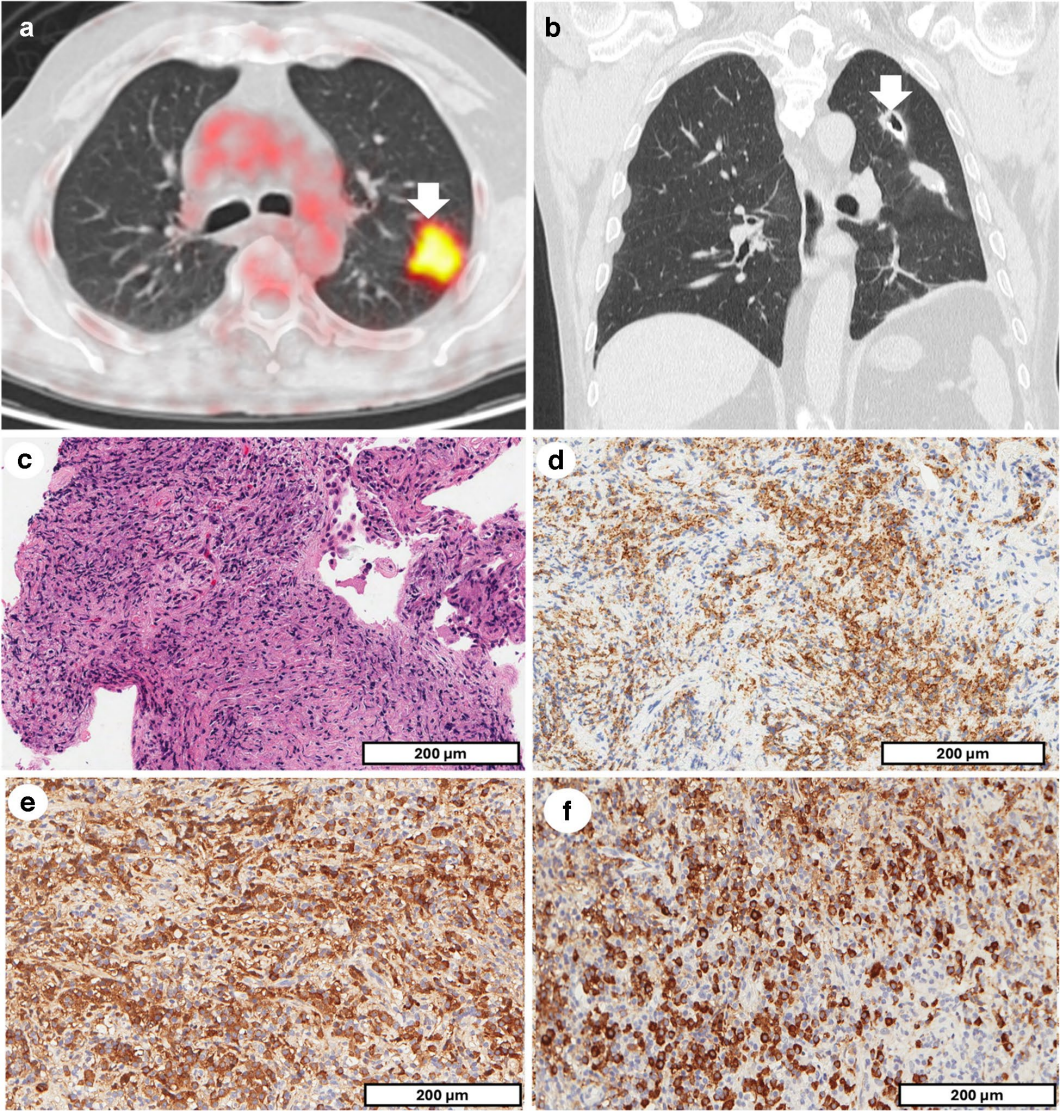
Case 2: Radiological progression and histopathological confirmation of IgG4-RD. The [18F]-FDG PET/CT showed a pulmonary consolidation in the left upper lobe with high metabolic activity (white arrow in **a**). Coronal reconstruction of the chest CT performed 2 years later, after surgery, showing two pulmonary nodules along the surgical suture, the upper one cavitated (white arrow in **b**). Histological analysis of the biopsied nodules showed an inflammatory infiltrate (**c**, scale bar: 200 µm), rich in plasma cells (CD138, **d**, scale bar: 200 µm). Immunohistochemical staining showed IgG-positive plasma cells (**e**, scale bar: 200 µm) with a marked predominance of IgG4-expressing cells (average of 74 IgG4-positive plasma cells per HPF); the IgG4⁺/IgG⁺ ratio was estimated at 55% by comparing counts across high-power fields (**f**, scale bar: 200 µm)

A 67-year-old male, former smoker, with a medical history of bronchial asthma and ischemic heart disease (including prior STEMI), underwent routine imaging in 2016 which revealed a 2.5-cm solitary pulmonary nodule in the left upper lobe that showed high FDG uptake on PET/CT (Fig. 2a). Given the concerning radiological features, a solitary, metabolically active pulmonary nodule in a smoker with underlying lung disease, the differential diagnosis at that time included primary lung carcinoma, granulomatous infection, and post-infectious scar tissue. Due to the high FDG uptake and clinical risk profile, the nodule was surgically resected. Histopathological analysis of the tissue showed extensive fibrosis and chronic inflammation, both subpleural and interstitial, accompanied by eosinophilia and bronchiectasis with abscess formation. The lesion also featured an epithelioid histiocytic wall, commonly associated with follicular bronchiolitis (FB). Additionally, histological evidence of past tuberculosis contributed to the substantial scarring observed in the lung tissue. In the years following the surgery, the patient experienced sporadic hemoptysis but did not undergo further diagnostic evaluation. A follow-up CT scan in 2022 revealed solid nodules along the surgical suture in the left lung, one of them cavitated (Fig. 2b) raising concerns about scar carcinoma, a recognized complication in patients with previous pulmonary infections, fibrosis, or surgery. Given the radiological data, the history of smoking, and prior lung disease, the primary differential diagnosis was scarring carcinoma. However, a biopsy of the nodules revealed a different diagnosis. Histopathological examination showed fibrosis with dense plasma cell infiltration (Fig. 2c, d). IHC demonstrated a significant increase in IgG4-positive plasma cells (Fig. 2e, f). Serum IgG4 levels were not available in this case, and no extrathoracic organ involvement was observed clinically or radiologically. These features were consistent with a probable diagnosis of IgG4-RD [2]. Following this diagnosis, the patient began corticosteroid therapy. After 3 months, his clinical and radiological condition improved significantly. Additionally, his episodes of hemoptysis were resolved, and overall lung function improved.

### Case 3

**Fig. 3:**
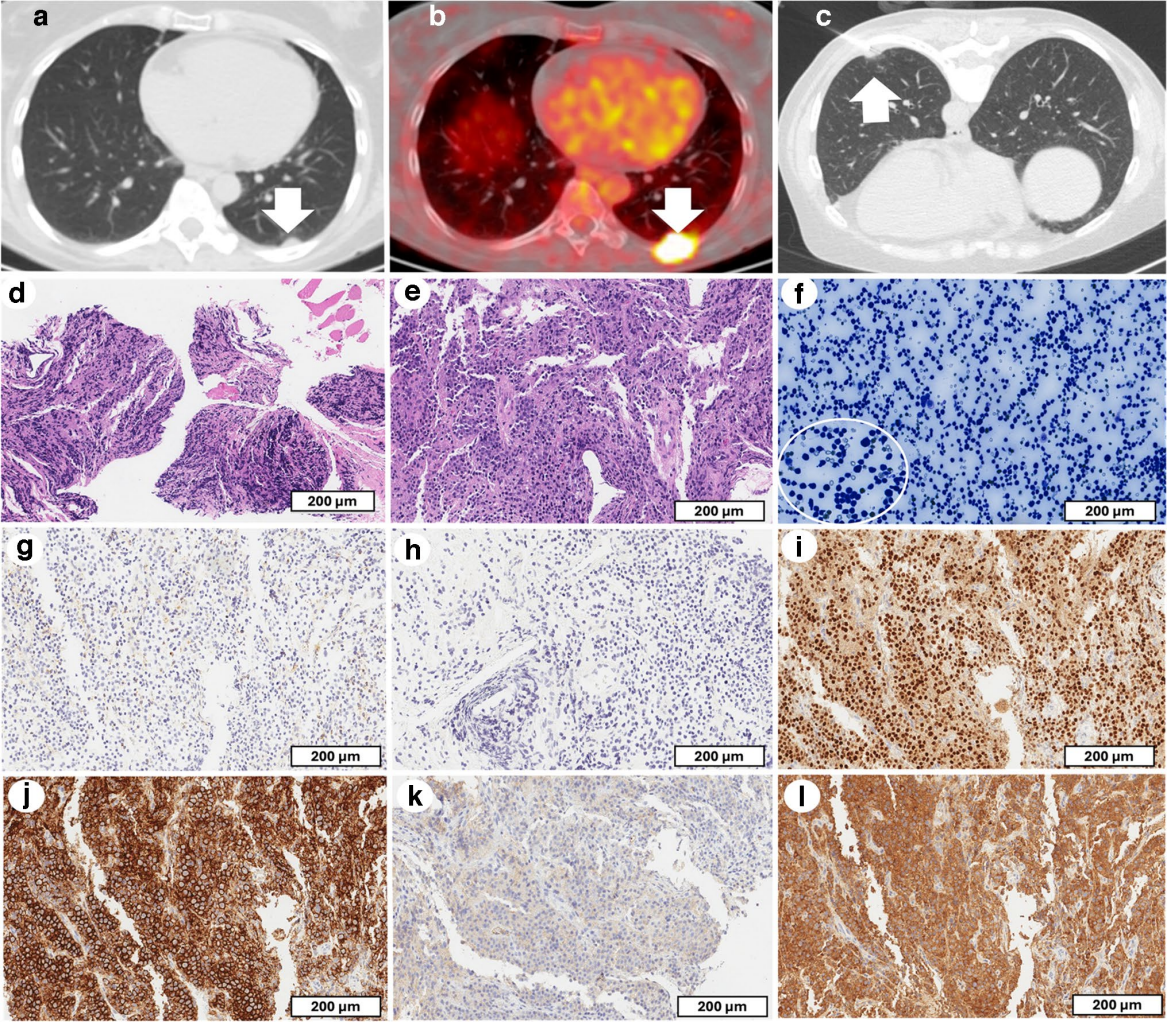
Case 3: Histopathological and immunohistochemical findings in lung tissue and pleural effusion. Axial chest CT with lung window demonstrating the presence of a subpleural nodule in the left lower lobe (white arrow in **a**). The same nodule showed high FDG uptake at the subsequent PET/CT (white arrow in **b**). The CT-guided biopsy of the nodule (white arrow, **c**). Microscopic evaluation detected extensive subpleural inflammatory infiltration within the lung parenchyma (**d**, scale bar: 300 µm), with dense aggregates of pleomorphic plasma cells within a fibrotic stroma (**e**, scale bar: 200 µm). The presence of plasma cells was also reported in the pleural effusion (**f**, scale bar: 200 µm). Cytokeratin and CD20 staining were negative (MNF116, **g**, scale bar: 200 µm; **h**: CD20, **h**, scale bar: 200 µm). Plasma cells were strongly positive for MUM-1 (**i**, scale bar: 200 µm) and CD138 (**j**, scale bar: 200 µm). Light chain analysis showed no detectable kappa staining (**k**, scale bar: 200 µm) and restricted lambda expression (**l**, scale bar: 200 µm)

This report discusses a 57-year-old female diagnosed with multiple myeloma (MM), with a focus on her pulmonary involvement that emerged several years after the initial diagnosis. The patient’s disease was first diagnosed in October 2015, when she presented with multiple areas of bone lytic lesions and a bone marrow biopsy revealing 50% plasma cell infiltration characterized by lambda light chain restriction. Following this, she underwent autologous stem cell transplantation in July 2016, which successfully reduced bone marrow plasma cell infiltration to 8%, as confirmed 6 months later. Despite achieving initial remission, the disease progressed in 2019, though remission was again achieved after further treatment. In December 2023, a subpleural nodule was identified at CT, and it also showed high FDG uptake at the subsequent PET/CT (Fig. 3a, b), suggesting pulmonary involvement. A transthoracic needle biopsy of the lesion was performed. Histopathological examination showed a prominent infiltration of atypical plasma cells throughout the lung parenchyma. Hematoxylin and eosin staining revealed dense aggregates of pleomorphic plasma cells embedded in a desmoplastic stroma, with predominant involvement of the subpleural regions (Fig. 3d, e). A predominance of plasma cells was also detected in the pleural effusion (Fig. 3f). These findings suggested an extramedullary manifestation of multiple myeloma. IHC confirmed the diagnosis, with plasma cells testing positive for MUM-1. Strong lambda light chain restriction was observed, ruling out kappa expression. Additionally, the absence of CD20 excluded B-cell lymphoma, and the lack of MNF116 expression helped exclude the possibility of epithelial malignancy (Fig. 3g–l).

### Case 4

**Fig. 4:**
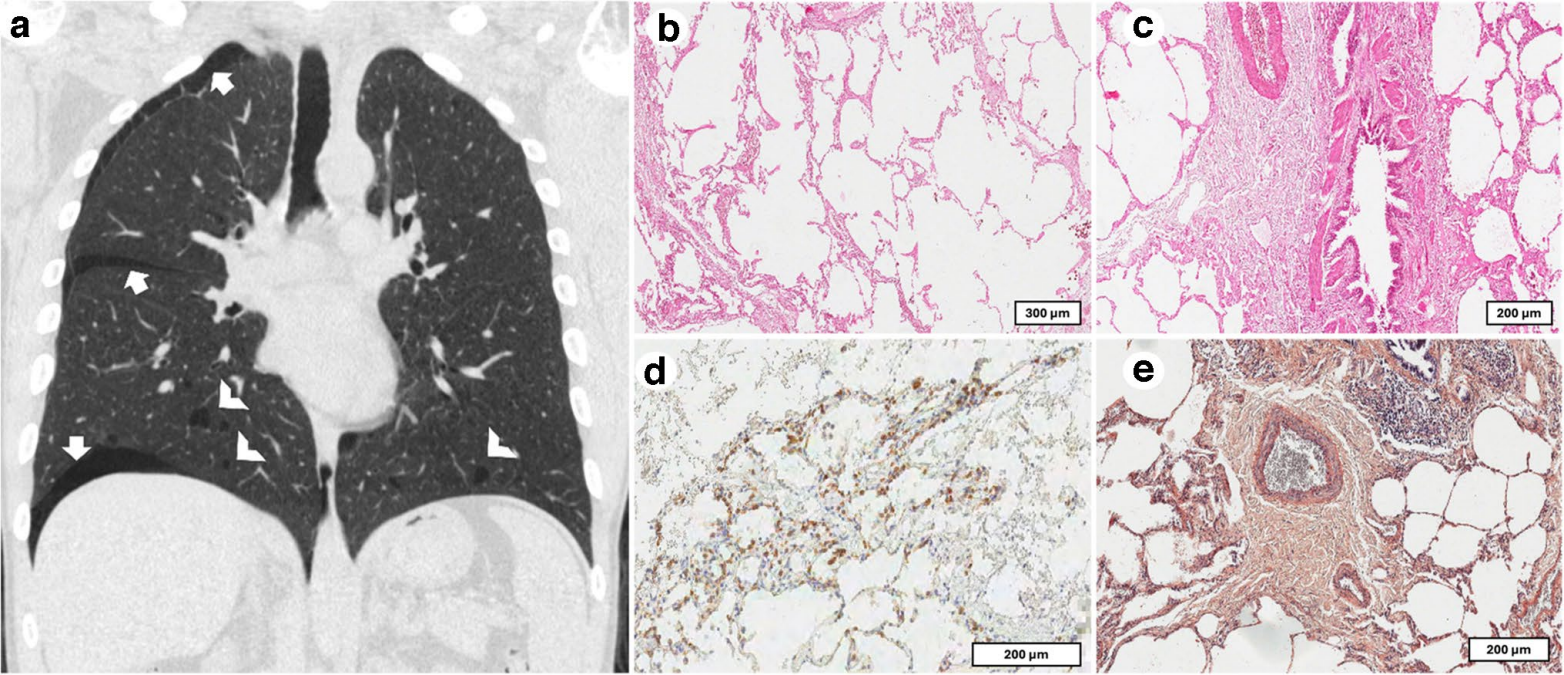
Case 4: Histopathological features of pulmonary alterations. Coronal reconstruction with the lung window demonstrating the pneumothorax (white arrows in **a**) and the bilateral cysts (white arrowheads in **a**). Histological examination showed cystic alveolar spaces (**b**, scale bar: 300 µm), with a mild inflammatory infiltrate composed of lymphocytes and plasma cells (**c**, scale bar: 200 µm). Small aggregates of plasma cells were also observed in the lung parenchyma (**d**, scale bar: 200 µm). Congo red staining showed the absence of apple-green birefringence in amorphous extracellular substance deposits, detected in peribronchial areas (**e**, scale bar: 300 µm)

A 34-year-old male, a lifelong non-smoker, presented with a complex clinical history including celiac disease and pigmented retinopathy. He had experienced bilateral pneumothorax on two separate occasions, 5 years apart. The patient also reported renal symptoms, including dysuria and polyuria, alongside a high-titer antinuclear antibody positivity of 1:1280. Following his second pneumothorax (Fig. 4a), the patient underwent video-assisted thoracoscopic surgery (VATS), during which tissue samples were obtained. Initial pathological evaluation diagnosed centrilobular emphysema. However, a subsequent review of the histological specimens at our center revealed different findings: alveolar cystic ectasis, mild lymphoplasmacytic infiltration (Fig. 4b, c), and small clusters of plasma cells (Fig. 4d). Notably, there was a deposition of amorphous material in the peribronchial region, which tested negative for amyloid upon Congo red staining and polarized light microscopy (Fig. 4e). Definitive confirmation with mass spectrometry was not performed in this case. Although the findings remain inconclusive due to the absence of confirmatory mass spectrometry, this case illustrates a frequent real-world scenario in which tissue limitations or unavailable molecular testing necessitate a diagnosis of exclusion.

## Diagnostic challenges

The presented cases highlight the diverse spectrum of plasma cell-associated lung disorders, underscoring the diagnostic challenges previously discussed. Plasma cell infiltration, as demonstrated in these cases, can vary from benign conditions like IgG4-RD to malignant neoplasms such as MM (Table 1). Other important entities must be considered in the differential diagnosis of plasma cell-rich lung lesions [3]. These include systemic connective tissue diseases (e.g., rheumatoid arthritis, systemic lupus erythematosus), immunodeficiency-related lymphoproliferative disorders (e.g., HIV-associated LPDs), and hematologic malignancies such as extranodal marginal zone lymphoma of mucosa-associated lymphoid tissue (MALT lymphoma) and lymphoplasmacytic lymphoma (LPL). The primary challenge for clinicians and pathologists is distinguishing between these conditions, given their often-overlapping clinical presentations, radiological findings, and histopathological features. Accurate differentiation is essential for guiding appropriate treatment strategies and improving patient outcomes.

**Table 1:** Differential diagnosis of plasma cell-related lung lesions

Condition	Clinical presentation	Imaging findings	Histopathology	IHC/markers	Key molecular/ancillary studies	Treatment	Prognosis
IgG4-related disease	Subacute onset; mild respiratory symptoms; lung nodules, pleural effusions	Nodules, ILD, adenopathy	Lymphoplasmacytic infiltrate, storiform fibrosis	↑ IgG4 +/IgG ratio; polyclonal light chains; CD138 +; Congo red −	Not typically required	Steroids, immunosuppression	Good with treatment, relapse possible
LCDD	Cough, dyspnea, cystic disease	Cysts, nodules, emphysema	Perivascular eosinophilic deposits	Monoclonal (kappa > *λ*); CD138 variable; Congo red −	Rare light chain rearrangement	Targeted therapy	Poor if untreated
MM/plasmacytoma	Dyspnea, systemic MM signs	Masses, lytic bone lesions	Monoclonal plasma cells	CD138 +; monoclonal light chains; Congo red −	FISH, IgH rearrangement	Chemotherapy, transplant	Variable
CTD-ILD	Cough, fatigue, extrapulmonary symptoms	Basal ILD, reticulation, traction bronchiectasis	Lymphoid infiltrates, fibrosis	Polyclonal plasma cells; mixed B/T cells; Congo red −	Not routinely required	Immunosuppressants	Variable, depends on subtype
Immunodeficiency disorders	Recurrent infections, lymphadenopathy	Adenopathy, nodules	Hyperplastic follicles, plasma cells	Polyclonal plasma cells; follicular hyperplasia; Congo red −	HIV test, flow cytometry	Treat underlying cause	Variable
MALT lymphoma	Cough, incidental finding, B symptoms	Nodular consolidations, air bronchograms	Centrocyte-like cells, lymphoepithelial lesions	CD20 +, Bcl-2 +; light chain restriction; Congo red −	IgH rearrangement, BIRC3-MALT1 fusion	Radiotherapy, rituximab	Indolent, good prognosis
LPL	Fatigue, IgM monoclonal gammopathy	Diffuse infiltrates, nodules	Small lymphocytes, plasma cells	IgM +; MYD88 mutation; monoclonal light chains; Congo red −	MYD88 L265P mutation	Chemotherapy ± rituximab	Indolent, may transform

*CD* cluster of differentiation; *FISH* fluorescence in situ hybridization; *IgH* immunoglobulin heavy chain; *ILD* interstitial lung disease; *CTD* connective tissue disease; *HIV* human immunodeficiency virus; *MALT* mucosa-associated lymphoid tissue; *LPL* lymphoplasmacytic lymphoma; *LCDD* light chain deposition disease; *MM* multiple myeloma

Pulmonary nodular lymphoid hyperplasia (PNLH), lymphoid interstitial pneumonia (LIP), and FB are not considered plasmacytic diseases per se. These conditions are best classified as reactive lymphoid proliferations, often occurring in association with autoimmune diseases or immunodeficiency. However, given their occasional abundance of plasma cells, they may enter the histologic differential diagnosis of plasma cell-rich lung lesions and therefore merit discussion in this context [4]. PNLH, for instance, typically appears as a solitary, well-defined pulmonary nodule that could be mistaken for malignancies on imaging and requires careful histopathological and immunohistochemical analysis to distinguish it from more aggressive lymphoproliferative disorders such as MALT lymphoma [5–7].

FB is characterized by the formation of lymphoid follicles within the walls of small airways, specifically bronchioles, and may present with mosaic attenuation, air trapping, or small airway thickening on CT [7]. This leads to narrowing or even complete obliteration of the bronchiolar lumen, resulting in obstructive lung disease [8]. The secondary features of FB can include organizing pneumonia, obstructive pneumonia, and the presence of intraluminal neutrophils, which add to the complexity of the disease. Immunohistochemically, CD20 and CD79a-positive B lymphocytes are found around the peribronchial regions, while CD3-positive T cells populate the alveolar interstitium [8]. One of the key histological differentiators of FB is its localization to the bronchioles, with well-formed lymphoid follicles around the airways, which restricts its involvement largely to airway structures. This localization contrasts with the more diffuse septal and parenchymal involvement seen in LIP.

On the other hand, LIP is a more diffuse condition, characterized by the widespread infiltration of lymphocytes into the interstitium of the lung, including the bronchovascular bundles and interlobular septa [9]. LIP typically shows bilateral ground-glass opacity (GGO) and thin-walled cysts in a basal or peribronchovascular distribution [7]. LIP also features the presence of lymphoid follicles with germinal centers, histiocytes, and macrophages, but these lymphoid aggregates are not restricted to the airways, as seen in FB. The involvement of the interlobular septa and alveolar structures in LIP often leads to disruption and inflammation, with features such as type II pneumocyte hyperplasia and, in some cases, cyst formation without marked fibrosis. Additionally, LIP can present loose, ill-defined epithelioid granulomas; the presence of interstitial or intra-alveolar giant cells further complicates the histopathological picture. Immunohistochemical markers in LIP are similar to FB, with CD20 and CD79a positivity in the peribronchial regions, but the more widespread involvement of lymphocytes, including CD3-positive T cells, in the alveolar interstitium is a distinguishing feature. LIP must be differentiated from conditions such as nonspecific interstitial pneumonia (NSIP), usual interstitial pneumonia (UIP), and hypersensitivity pneumonitis (HP) [9]. NSIP lacks the lymphoid follicles seen in LIP and typically presents with either loose or dense fibrosis. UIP is characterized by fibroblastic foci and honeycomb changes; while these features are more typical of UIP, longstanding cases of LIP and other chronic inflammatory lung diseases can also develop severe fibrosis and even honeycombing, making histologic distinction challenging in advanced disease. HP, especially the non-fibrosing variant, may show organizing pneumonia but lacks the lymphoid hyperplasia seen in LIP. In contrast, FB must be differentiated from MALT lymphoma, where monoclonal populations of B cells and Dutcher bodies may be present, findings which are not seen in FB or LIP. An increase in lymphocytes, especially CD8-positive T cells, can be observed in bronchoalveolar lavage samples, although the presence of clonality in lymphocytes may suggest progression to a neoplastic process, such as MALT lymphoma, in more severe cases. Infectious etiologies, such as the *Legionella pneumophila* infection, have also been associated with FB, emphasizing the need for thorough clinical and microbiological evaluations when diagnosing these lymphoid proliferations.

**Fig. 5:**
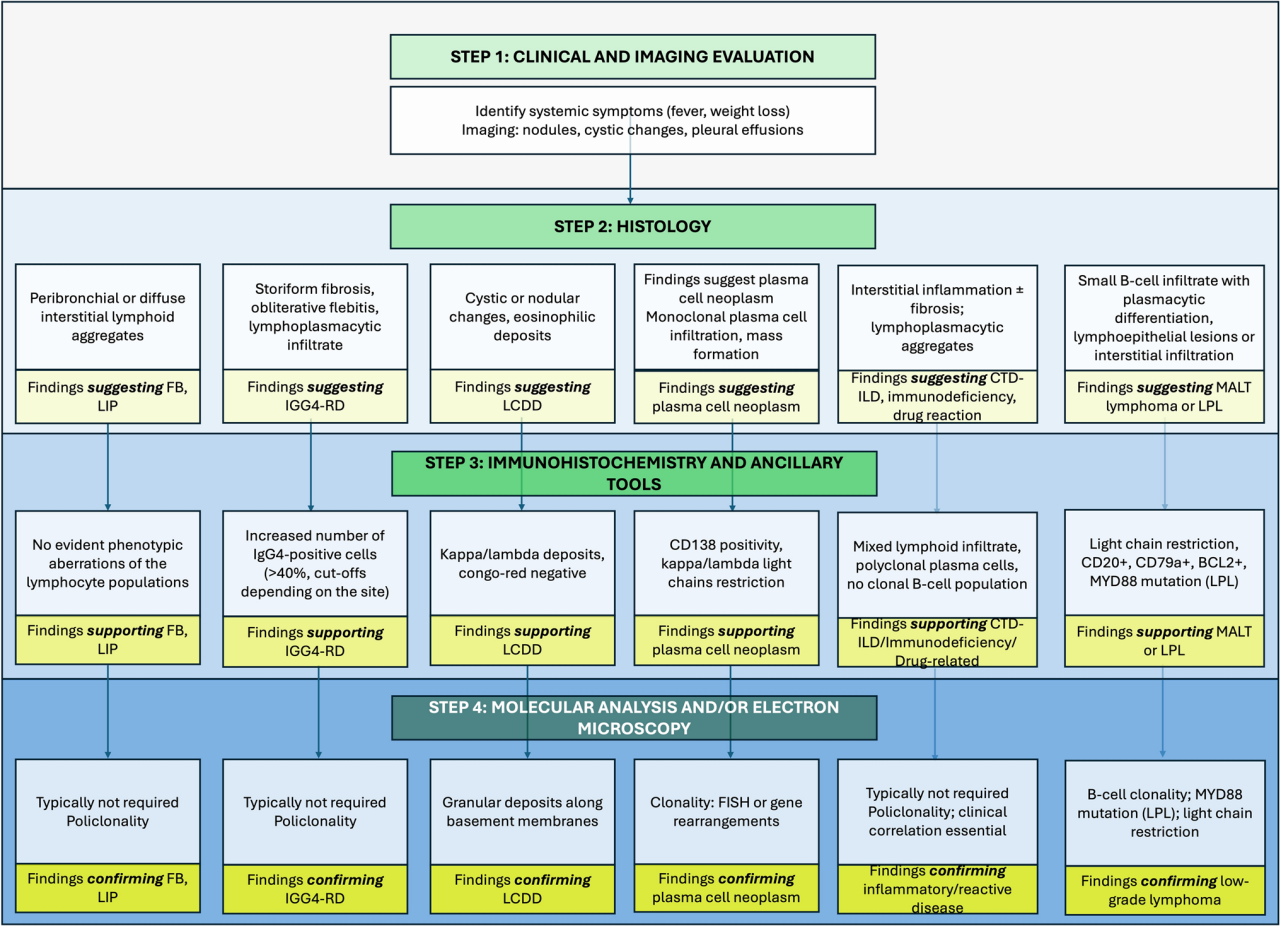
Integrated diagnostic approach to pulmonary plasma cell disorders. The flowchart outlines a diagnostic strategy incorporating clinical, radiological, histopathological, and immunohistochemical assessments. Key elements include evaluating plasma cell distribution, clonality, fibrosis, necrosis, and protein deposition, complemented by serological and molecular studies for definitive classification

Taken together, the four presented cases highlight the complexity of plasma cell-rich lung disorders. Case 1 and Case 2 emphasize the histological thresholds needed for diagnosing IgG4-RD, while Case 3 represents a clear example of a clonal neoplasm with systemic features. Case 4 illustrates the difficulties in confirming LCDD, especially in the absence of advanced proteomic studies. These examples demonstrate how a diagnostic algorithm (Fig. 5) can be applied to stratify cases along the reactive, fibroinflammatory, and neoplastic spectrum, promoting more consistent diagnostic reasoning and therapeutic planning.

Importantly, not all plasma cell-rich lesions yield a definitive diagnosis despite exhaustive histological evaluation. As demonstrated in Case 4, tissue sampling limitations or the unavailability of specialized tools such as mass spectrometry can leave certain entitiesì presumptive rather than confirmed. Rather than diminish their educational value, these ambiguous cases emphasize the need for a systematic diagnostic approach and reinforce the critical role of multidisciplinary correlation in challenging scenarios.

### IgG4-RD

IgG4-RD is a systemic fibroinflammatory condition that can affect virtually any organ in the body, with common sites including the pancreas, salivary glands, and orbit [10]. However, pulmonary involvement, as seen in the present case, can be challenging to diagnose due to its rarity and the nonspecific nature of early symptoms such as pleuritis, lung nodules, and interstitial changes [11]. Interstitial lung disease is another possible presentation, leading to diffuse or patchy infiltrates resembling other types of interstitial pneumonia, such as idiopathic pulmonary fibrosis [12]. Pleural involvement is less common, occurring in only 4.6% of cases, but may present as pleural thickening or effusion [12, 13]. A study by Yoh Zen et al. in the American Journal of Surgical Pathology identified four distinct pulmonary patterns associated with IgG4-RD: solid nodular lesions, broncho-vascular involvement, alveolar interstitial involvement, and round GGO. Although not all of these radiologic patterns were represented in the cases presented in this manuscript, the solid nodular form and fibrotic changes observed in Cases 1 and 2 align with the nodular and broncho-interstitial patterns described. These radiologic patterns can mimic infections, malignancy, or chronic interstitial pneumonias, thus necessitating histologic correlation and careful exclusion of alternate etiologies [12, 13].

Histopathologically, IgG4-RD is defined by a dense lymphoplasmacytic infiltrate rich in IgG4-positive plasma cells, storiform fibrosis, and often obliterative phlebitis [10, 14, 15]. In pulmonary involvement, inflammation frequently affects bronchovascular bundles, interstitial septa, and pleura, with classic storiform fibrosis often focal or absent, particularly in small biopsy samples. Obliterative phlebitis and arteritis are generally more prominent in the lung compared to other organs [16]. A hierarchical classification system defines definitive IgG4-RD as the presence of at least two of the three major histologic features; probable cases display only one, and possible cases lack typical histology but are supported by clinical and radiologic context [2, 10, 14]. This approach is crucial for interpreting limited pulmonary specimens [17].

#### Differential diagnosis of IgG4-RD

**Fig. 6:**
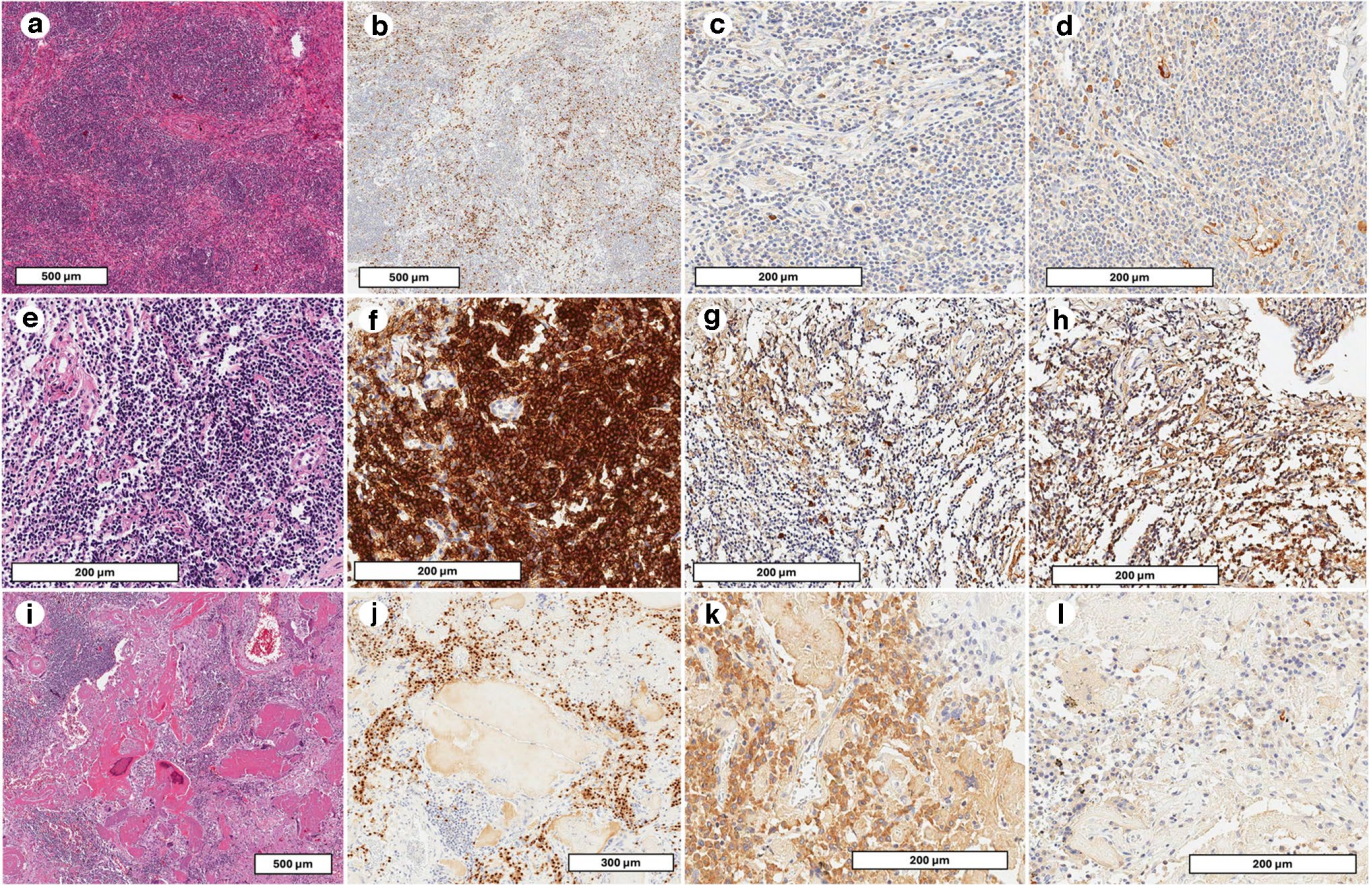
Histologic features of key differential diagnoses for IgG4-RD. This panel illustrates three key entities that may mimic IgG4-RD both histologically and clinically. Seventy-six-year-old male with persistent dry cough, dysphonia, and weight loss since March 2019. Chest CT with contrast revealed a lesion in the right upper lobe. Fine-needle biopsy showed a dense lymphoid infiltrate composed of small lymphocytes (**a**, scale bar: 500 µm). IHC demonstrated CD20-positive B cells (**b**, scale bar: 500 µm), without clear light chain restriction (**c**, **d**, immunohistoichemistry for kappa and lambda chains, scale bar: 200 µm). However, polymerase chain reaction (PCR)-based analysis confirmed IGH gene rearrangement, supporting B-cell clonality and the diagnosis of extranodal marginal zone lymphoma. Seventy-five-year-old male, former smoker with COPD, previously employed as a warehouse worker with potential exposure to airborne irritants. No allergies or chronic medications; otherwise, unremarkable history. In July 2023, HRCT for recurrent bronchitis revealed a 2–3 cm consolidation in the superior segment of the right lower lobe, without effusion or lymphadenopathy. Histology showed a well-circumscribed nodular infiltrate of mature plasma cells in dense sheets, with scattered lymphocytes, reactive follicles, and focal stromal fibrosis (**e**, scale bar: 200 µm). No necrosis, atypia, or invasion. MUM1 was diffusely positive (**f**, scale bar: 200 µm). Kappa (**g**, scale bar: 200 µm) and lambda (**h**, scale bar: 200 µm) showed a polyclonal distribution, supporting the diagnosis of plasma cell granuloma. Seventy-four-year-old male with centrilobular emphysema. Chest CT for lung cancer screening revealed a 4.5 cm solid nodule in the left lower lobe, with no lymphadenopathy or effusion. Histology showed dense plasma cell infiltrates with focal stromal deposits of eosinophilic amorphous material (**i**, scale bar: 300 µm), Congo red-positive with apple-green birefringence under polarized light. MUM1 highlighted plasma cells (**j**, scale bar: 300 µm), and IHC demonstrated kappa light chain restriction (**k**, **l**, immunohistoichemistry for kappa and lambda chains, scale bar: 200 µm), confirming a diagnosis of pulmonary AL kappa-restricted amyloidoma

The histological and immunohistochemical findings in this case underscore the importance of identifying “hot spots” where lymphoplasmacytic infiltration is most prominent [14]. Standard practice involves counting IgG4-positive plasma cells in these areas across multiple high-power fields, ensuring accurate disease assessment. It is crucial to differentiate IgG4-RD from other conditions with similar histopathological features, such as sarcoidosis, vasculitis (e.g., granulomatosis with polyangiitis), and lymphomas. Sarcoidosis typically presents with well-formed, non-necrotizing granulomas, often without significant plasma cell infiltration. In contrast, IgG4-RD shows a lymphoplasmacytic infiltrate, storiform fibrosis, and obliterative phlebitis, with IgG4-positive plasma cells in high numbers. Vasculitis like granulomatosis with polyangiitis often displays necrotizing granulomas and fibrinoid necrosis of vessel walls, which are absent in IgG4-RD. Lymphomas, especially MALT lymphoma or lymphoplasmacytic lymphoma, may histologically mimic IgG4-RD due to dense infiltrates and plasmacytic differentiation; however, these entities usually demonstrate light chain restriction, monoclonal B-cell populations (detected via flow cytometry or molecular studies), and may harbor MYD88 mutations. MYD88 mutations, particularly the L265P variant, are most commonly associated with lymphoplasmacytic lymphoma and may serve as a useful molecular marker to distinguish this entity from other small B-cell lymphomas and from plasma cell neoplasms [18]. Therefore, ancillary studies including light chain IHC/ISH, clonality assessment, and flow cytometry are essential for accurate classification [14]. In some cases, granulomas may occasionally coexist with IgG4-RD; their presence generally excludes the diagnosis unless other classic features of IgG4-RD are evident. In this context, plasma cell granulomas (PCG) or inflammatory pseudotumors (IPT) deserve special mention (Fig. 6). These lesions can closely mimic IgG4-RD histologically, displaying dense plasma cell-rich infiltrates, fibrosis, and even phlebitis, but lack diagnostic thresholds immunohistochemically (e.g., low IgG4/IgG ratios) and serologically (e.g., normal serum IgG4) [1]. Unlike true IgG4-RD, they are usually polyclonal and not associated with systemic involvement [1].

In certain plasma cell-rich lesions, particularly those associated with amyloid deposits, a confluent lymphoid infiltrate may raise suspicion for an underlying B-cell lymphoma with plasmacytic differentiation. These cases are diagnostically complex because plasma cells may demonstrate light chain restriction, while the B-cell component may not display overt aberrancy by IHC, especially in MALT lymphomas, which are typically CD5-negative and CD10-negative. In such settings, flow cytometry becomes an essential tool, as it enables the detection of clonal B-cell populations that are otherwise immunophenotypically silent. The utility of flow cytometry is particularly evident when plasmacytic differentiation obscures conventional histologic interpretation. Similarly, molecular analysis, such as IGH rearrangement studies, can provide confirmatory evidence of clonality. However, IGH gene rearrangement analysis identifies the presence of a clonal immunoglobulin-producing population but does not discriminate between clonal B lymphocytes and neoplastic plasma cells, as both cell types arise from the same lineage. Consequently, a monoclonal IGH rearrangement may be observed in either B-cell lymphomas or in plasma cell neoplasms [18]. In this context, multiparameter flow cytometry represents the preferred modality for evaluating B-cell clonality, as it enables detailed immunophenotypic characterization, including surface antigen profiling and assessment of light chain restriction. However, flow cytometric analysis necessitates viable, unfixed tissue, typically fresh or appropriately preserved, which may not be available when lymphoid or plasma cell malignancy is not clinically suspected at the time of biopsy. In cases where initial pathology is inconclusive and clinical suspicion for a lymphoproliferative or plasma cell disorder persists, a repeat biopsy with dedicated tissue procurement for flow cytometry should be strongly considered. This approach is particularly valuable for distinguishing between indolent B-cell lymphomas with plasmacytic differentiation and bona fide plasma cell neoplasms, especially when immunohistochemical and molecular findings alone are insufficient to establish lineage specificity.

Moreover, IgG4-RD shares histopathological overlap with multicentric Castleman disease [20], another lymphoproliferative disorder characterized by polyclonal plasma cell infiltration, fibrosis, and elevated IgG4 levels in some cases.

IgG4-RD is most commonly observed in middle-aged to older adults, with a slight male predominance, as in this case [10, 11]. The pathogenesis is thought to involve chronic antigenic stimulation, leading to a breach of immune tolerance. This process stimulates an oligoclonal expansion of IgG4-positive plasmablasts and CD4 + T cells, contributing to the characteristic fibrosis and plasma cell infiltration seen in affected tissues [10, 21]. While IgG4 is generally considered an anti-inflammatory immunoglobulin, overproduction and excessive infiltration of IgG4-positive cells lead to pathological fibrosis and tissue damage [10, 21].

A key diagnostic challenge in plasma cell-rich lung lesions lies in distinguishing reactiveness from neoplastic infiltrates (Table 1) [22, 23]. Reactive infiltrates, typically polyclonal, may arise in infections, autoimmune diseases, or connective tissue disorders and express both kappa and lambda light chains. In contrast, neoplastic conditions, such as MM, plasmacytoma, or lymphomas, exhibit monoclonal plasma cells with light chain restriction. IHC and in situ hybridization are essential to demonstrate this. IHC and molecular studies, including clonal immunoglobulin gene rearrangement analysis and fluorescence in situ hybridization (FISH), are invaluable tools for differentiating these conditions [24]. FISH can be employed to detect chromosomal aberrations commonly associated with plasma cell neoplasms, such as IGH (14q32) translocations, deletions involving chromosome 13q, or amplifications of 1q21, which support a diagnosis of multiple myeloma or plasmacytoma in appropriate histologic settings. In cases suspicious for MALT lymphoma, FISH may identify rearrangements involving BIRC3-MALT1 or IGH, further supporting clonality [3].

Recent studies indicate that IgG4-RD may function as a paraneoplastic phenomenon, particularly in malignancies such as pancreatic, pulmonary, and gastric carcinomas [25]. In these cases, increased IgG4 serum levels and histopathological hallmarks of IgG4-RD can coexist with or even precede the detection of an underlying neoplasm. The precise relationship between IgG4-RD and tumorigenesis remains under investigation. Chronic inflammation mediated by IgG4-positive plasma cells may play a role in oncogenesis or, alternatively, reflect an immune response triggered by the neoplastic process [26].

Finally, in regions with endemic parasitic infections, parasitic diseases such as schistosomiasis, filariasis, or toxocariasis should be considered as differential diagnoses, particularly in patients with a relevant travel or exposure history. Both diseases can present with eosinophilia, lymphoplasmacytic infiltration, and chronic tissue damage leading to fibrosis [27, 28]. The identification of parasitic eggs or larvae within the tissue is critical for diagnosing parasitic infections.

## Plasma cell neoplasms and associated disorders

A central consideration in the evaluation of plasma cell-rich lung lesions is the possibility of an underlying plasma cell neoplasm, most notably multiple myeloma (MM) and solitary plasmacytoma [19].

These conditions represent the neoplastic end of the plasma cell disease spectrum and may manifest as pulmonary masses, nodules, or diffuse infiltrates. While MM is a systemic disease defined by clonal bone marrow plasma cells ≥ 10% and associated end-organ damage (e.g., anemia, hypercalcemia, lytic bone lesions), plasmacytoma refers to a localized clonal proliferation of plasma cells without bone marrow involvement or systemic features. In cases where no prior hematologic diagnosis exists, a pulmonary biopsy showing a monoclonal plasma cell infiltrate should prompt a comprehensive systemic workup. This includes serum protein electrophoresis (SPEP), immunofixation, free light chain assay, and bone marrow biopsy to assess for systemic disease. Imaging studies such as PET/CT or skeletal survey may help identify occult osseous lesions. IHC (e.g., CD138, kappa/lambda light chains) and molecular studies (e.g., FISH for IGH rearrangement, 13q deletion, or 1q gain) further support clonality and aid in classification. Importantly, conditions such as LCDD and AL-type amyloidosis often reflect underlying plasma cell dyscrasias, even in the absence of overt lung involvement, and must be carefully evaluated in this context. Although plasma cell neoplasms rarely involve the lungs directly, when they do, they can manifest as plasmacytomas, masses of neoplastic plasma cells in the lung parenchyma or pleura. In MM, for instance, pulmonary involvement typically arises in the context of disseminated disease, with plasmacytomas being the predominant form of lung infiltration [4, 5]. Patients often complain of respiratory symptoms such as dyspnea or a cough, along with systemic manifestations of MM, including bone lesions, hypercalcemia, and renal dysfunction. Histopathological evaluation typically reveals monoclonal plasma cells, which can be confirmed through immunohistochemical staining for markers such as CD138 and light chain restriction (kappa or lambda). Molecular studies, including FISH, can further identify genetic abnormalities characteristic of MM [4, 5].

MALT lymphoma and LPL are indolent B-cell lymphomas that can present with pulmonary involvement and are often considered in the differential diagnosis of plasma cell-rich infiltrates. MALT lymphoma typically presents as nodular or consolidative lesions in the lung and histologically shows small B cells, marginal zone cells, and plasmacytic differentiation, often with lymphoepithelial lesions. LPL, in contrast, involves the lung less frequently and is usually associated with IgM paraproteinemia and bone marrow involvement. IHC and molecular studies, including detection of monoclonality and MYD88 mutations, help distinguish these entities [18].

## LCDD in the lung: a pulmonary manifestation of plasma cell neoplasms

LCDD is a rare systemic disorder characterized by the deposition of monoclonal immunoglobulin light chains, most commonly of the kappa subtype, in various organs. While the kidneys are most frequently affected, the lungs can also be involved, though less commonly [29]. In LCDD, pulmonary involvement typically manifests as interstitial or nodular light chain deposits, often associated with cystic lung disease and emphysematous changes [30]. Patients may present with nonspecific respiratory symptoms such as cough, dyspnea, and, in some cases, oxygen desaturation [30]. The pathogenesis of lung involvement in LCDD is unclear, and no single genetic mutation predicts its occurrence [30]. LCDD typically affects individuals in their 50 s and 60 s, with a slight male predominance, and is commonly associated with underlying plasma cell neoplasms like MM [30].

LCDD and AL-type amyloidosis both involve the deposition of monoclonal immunoglobulin light chains but differ histologically and ultrastructurally [31]. LCDD shows non-fibrillar, PAS-positive, eosinophilic nodular deposits that are Congo red-negative, in contrast to amyloidosis, which is Congo red-positive and birefringent under polarized light due to its β-pleated sheet configuration [29]. Electron microscopy in LCDD reveals granular electron-dense material, while amyloidosis exhibits fibrillar deposits [32]. Mass spectrometry, especially LC–MS/MS following laser microdissection, is the current gold standard for definitive typing. Stains like Masson trichrome and sulfated alcian blue may aid initial differentiation [33].

MM can also cause pulmonary involvement, either through plasmacytomas or diffuse parenchymal infiltration, but systemic findings such as bone lesions and hypercalcemia help differentiate it from LCDD [34]. Other conditions, including monoclonal gammopathy of renal significance, may present with pulmonary light chain deposition, though primary pathology is typically renal [35]. Additionally, LPL and LIP should be considered, particularly when nodular lung lesions are present [36]. A biopsy with immunohistochemical studies, SPEP, and bone marrow biopsy is essential to distinguish these conditions from LCDD [37]. Moreover, while IgG4-RD and LCDD both involve plasma cell infiltration of the lung, they are pathophysiologically distinct and differential diagnosis can be challenging [38].

## Pulmonary amyloidosis: AL-type and beyond

Amyloidosis is a systemic or localized disorder characterized by extracellular deposition of misfolded protein in β-pleated sheet conformation. In the lungs, amyloid can be present in one of three patterns: tracheobronchial, nodular, or diffuse alveolar-septal amyloidosis [39]. Pulmonary involvement may be isolated or part of a systemic amyloid disease. The most common type of amyloid in the lung is AL (light-chain) amyloidosis, associated with plasma cell dyscrasias such as MM or monoclonal gammopathy of undetermined significance (MGUS) [39]. Figure 6 includes an illustrative example of AL-type pulmonary amyloidosis as part of the differential diagnosis of plasma cell-rich lung lesions. Histologically, amyloid appears as amorphous eosinophilic extracellular material that is Congo red-positive and exhibits apple-green birefringence under polarized light. Mass spectrometry-based proteomic typing has become a contemporary practice and is essential for diagnosis and subtype classification (e.g., AL, AA, ATTR), as each subtype has distinct clinical implications, prognostication, and treatment protocols [33, 39].

## Crystal-storing histiocytosis (CSH)

Another rare pulmonary manifestation of plasma cell and lymphoproliferative disorders is CSH [40, 41]. This condition is characterized by the accumulation of histiocytes with abundant eosinophilic cytoplasm containing intracytoplasmic crystalline immunoglobulin inclusions, typically kappa light chains. It almost always occurs in association with an underlying plasma cell neoplasm or a B-cell lymphoproliferative disorder [40, 41]. In the lung, CSH may present as solitary or multiple nodules, mass-like lesions, or interstitial infiltrates. Recognition is important, as the histologic features may mimic other histiocytic or granulomatous diseases, and identification of the underlying clonal disorder is critical for appropriate management.

## Conclusion

Given the broad spectrum of plasma cell-related lung conditions, from benign reactive infiltrates to malignant neoplasms and deposition diseases, a thorough diagnostic approach is essential (Fig. 5). Accurate diagnosis relies on integrating clinical, radiological, and histopathological data, supported by ancillary studies such as IHC, clonality testing, and, in select cases, molecular or proteomic techniques. Identifying the correct etiology informs management strategies and improves patient outcomes. A combination of clinical, radiological, and histological data is required to ensure an accurate diagnosis [6]. Histopathological examination remains the cornerstone of diagnosis, often supplemented by IHC and, when necessary, molecular studies to confirm clonality and identify genetic abnormalities [6]. In suspected cases of IgG4-RD, LCDD, or plasma cell neoplasms, tissue sections from the areas of the most significant plasma cell infiltration or deposition must be carefully evaluated [7]. For IgG4-RD, counting IgG4-positive cells in three high-power fields is recommended [8], while IHC for light chain restriction and Congo red staining is essential in diagnosing LCDD and amyloidosis [9]. Molecular studies, such as FISH or gene rearrangement tests, may be necessary to confirm plasma cell neoplasms [6]. The integration of flow cytometry and molecular diagnostics plays a critical role in resolving complex or overlapping features in plasma cell-rich lung lesions. Recognizing when to escalate to these techniques is essential for diagnostic accuracy and optimal patient care.

In conclusion, the integration of a structured algorithm, as proposed in Fig. 5, may aid pathologists and clinicians in navigating the often-overlapping features of these conditions, particularly in settings where clinical, histological, and molecular data must be weighed together for accurate diagnosis.

## Data Availability

Data sharing is not applicable to this article, as no datasets were generated or analyzed during the current study.
